# Repurposing Benztropine, Natamycin, and Nitazoxanide Using Drug Combination and Characterization of Gastric Cancer Cell Lines

**DOI:** 10.3390/biomedicines11030799

**Published:** 2023-03-06

**Authors:** Eduarda Ribeiro, Diana Araújo, Mariana Pereira, Bruna Lopes, Patrícia Sousa, Ana Catarina Sousa, André Coelho, Alexandra Rêma, Rui Alvites, Fátima Faria, Cláudia Oliveira, Beatriz Porto, Ana Colette Maurício, Irina Amorim, Nuno Vale

**Affiliations:** 1 OncoPharma Research Group, Center for Health Technology and Services Research (CINTESIS), Rua Doutor Plácido da Costa, 4200-450 Porto, Portugal; 2 CINTESIS@RISE, Faculty of Medicine, University of Porto, Alameda Professor Hernâni Monteiro, 4200-319 Porto, Portugal; 3 Departamento de Patologia e Imunologia Molecular, ICBAS—School of Medicine and Biomedical Sciences—University of Porto (UP), Rua de Jorge Viterbo Ferreira 228, 4050-313 Porto, Portugal; 4 Institute for Research and Innovation in Health (i3S), Universidade do Porto, Rua Alfredo Allen 208, 4200-135 Porto, Portugal; 5 Departamento de Clínicas Veterinárias, ICBAS—School of Medicine and Biomedical Sciences—University of Porto (UP), Rua de Jorge Viterbo Ferreira, n° 228, 4050-313 Porto, Portugal; 6 Centro de Estudos de Ciência Animal (CECA), Instituto de Ciências, Tecnologias e Agroambiente da Universidade do Porto (ICETA), Rua D. Manuel II, Apartado 55142, 4051-401 Porto, Portugal; 7 Associate Laboratory for Animal and Veterinary Sciences (AL4AnimalS), 1300-477 Lisbon, Portugal; 8 Laboratório de Citogenética, ICBAS—School of Medicine and Biomedical Sciences—University of Porto (UP), Rua de Jorge Viterbo Ferreira, n° 228, 4050-313 Porto, Portugal; 9 Institute of Molecular Pathology and Immunology, University of Porto (IPATIMUP), Rua Júlio Amaral de Carvalho 45, 4200-135 Porto, Portugal; 10 Department of Community Medicine, Health Information and Decision (MEDCIDS), Faculty of Medicine, University of Porto, Rua Doutor Plácido da Costa, 4200-450 Porto, Portugal

**Keywords:** benztropine, natamycin, nitazoxanide, gastric cancer, repurposing drugs

## Abstract

Gastric cancer (GC) ranked as the fifth most incident cancer in 2020 and the third leading cause of cancer mortality. Surgical prevention and radio/chemotherapy are the main approaches used in GC treatment, and there is an urgent need to explore and discover innovative and effective drugs to better treat this disease. A new strategy arises with the use of repurposed drugs. Drug repurposing coupled with drug combination schemes has been gaining interest in the scientific community. The main objective of this project was to evaluate the therapeutic effects of alternative drugs in GC. For that, three GC cell lines (AGS, MKN28, and MKN45) were used and characterized. Cell viability assays were performed with the reference drug 5-fluororacil (5-FU) and three repurposed drugs: natamycin, nitazoxanide, and benztropine. Nitazoxanide displayed the best results, being active in all GC cells. Further, 5-FU and nitazoxanide in combination were tested in MKN28 GC cells, and the results obtained showed that nitazoxanide alone was the most promising drug for GC therapy. This work demonstrated that the repurposing of drugs as single agents has the ability to decrease GC cell viability in a concentration-dependent manner.

## 1. Introduction

Human GC is the fifth most predominant cancer worldwide and the third leading cause of cancer-related death, infection with *Helicobacter pylori* being the major risk factor for this disease [1,2]. The incidence and mortality of GC have markedly decreased in recent decades worldwide, mainly in developed countries. However, in Eastern Europe, East Asia, and Central and South America, the incidence of GC remains high [3]. In addition, high-fat, high-salt, and high-nitrogen diets; Epstein–Barr virus (EBV); genetic factors; pre-malignant stomach lesions; and tobacco have been reported as risk factors for GC [4,5].

For advanced stage GC, combination therapy of surgery, chemotherapy, radiotherapy, and targeted therapy would benefit patients more. Chemotherapy is the principal clinical treatment method, but the rise of drug resistance limits its efficacy and may lead to treatment failure [6]. The drug 5-FU is considered the standard chemotherapy regimen [7]. This molecule is an analogue of uracil with an atom of fluorine at the C-5 position instead of hydrogen, preventing uracil from being incorporated into DNA during the “S” phase of the cell cycle, which stops normal development and cell division [8]. Despite several advantages, the clinical use of 5-FU has been limited due to the development of chemoresistance to the drug [9,10,11].

Despite the advancing research and recent progress in drug therapy treatment of solid tumors, the large number of cases and deaths in cancer patients is still a major health problem [12]. Drug repurposing aims to provide existing Food and Drug Administration-approved (FDA-approved) drugs new or additional indications. Currently, the profile of drug reuse has increased [13], since the pharmacokinetics, pharmacodynamics, toxicities, dosing schemes, and mechanisms of action of the drugs are already known [14,15]. Many non-oncology drugs act as anticancer agents by stopping proliferation and inducing cell death, as revealed through in vitro and in vivo studies or clinical trials [16,17,18].

**Figure 1:**
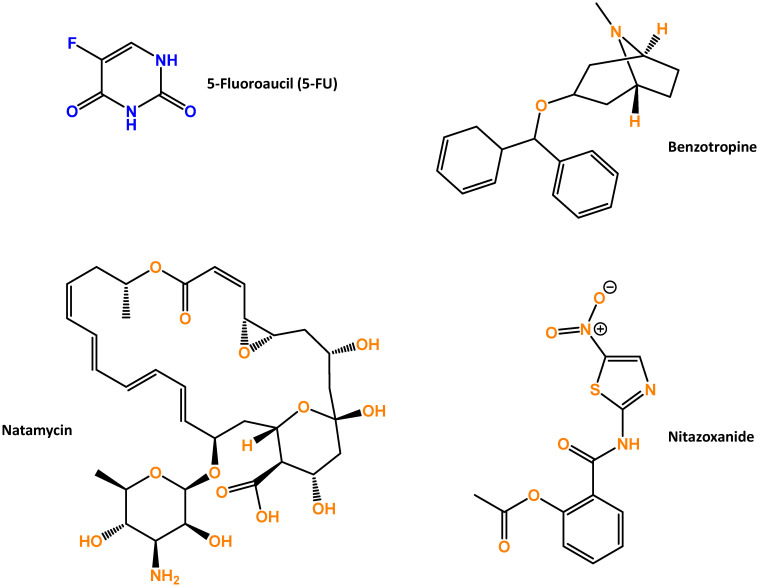
The chemical structures of drugs used in this project.

Natamycin (NAT) (Figure 1) is a polyene antibiotic used for treating superficial fungal infections because of its lack of resistance development and its broad spectrum of activity. NAT applies its fungal action by binding to ergosterol on the fungal cell membrane [19]. A recent study showed that NAT suppresses the enzyme base excision repair (BER) in prostate cancer, leading to a reduction in tumor cell proliferation. This enzyme is over-expressed in several types of cancer, including GC [20,21].

Nitazoxanide (NTZ) (Figure 1) is an FDA-approved antimicrobial agent used in parasitic intestinal infection treatment due to its repressive effect on pyruvate–ferredoxin–oxidoreductase, which is fundamental for the anaerobic metabolism of parasites [22,23]. However, NTZ also displays significant activity against cancer [24,25].

Benztropine (BZT) (Figure 1) is an anticholinergic drug currently usedr the treatment of Parkinson’s disease and dystonia [26]. BZT is known to improve Parkinson’s symptoms by the selective inhibition of dopamine transporters (DAT) and also presents an affinity to histamine and muscarine receptors [27,28,29].

The main aim of this investigation was to evaluate the therapeutic effects of repurposed drugs in GC. Therefore, the first part of this investigation focused on the morphology and biochemical, molecular, and genetic characterization of the three GC cell lines (AGS, MKN28, and MKN45); then, [3-(4,5-dimethylthiazol-2yl)-2,5-diphenyl tetrazolium bromide] (MTT) assays were performed concerning the reference drug 5-FU and the three additional repurposed drugs, namely, NAT, NTZ, and BZT (Figure 1).

## 2. Materials and Methods

Three human GC cell lines (AGS, MKN28, and MKN45), originally obtained from Biobank-i3s (Institute for Research & Innovation in Health), were used. All cell lines were originated from gastric carcinoma but harbor different genetics backgrounds.

### 2.1. Cell Lines and Cell Culture Conditions

Cells were cultured in RPMI-1640 medium (Gibco^®^, Grand Island, NY, USA) supplemented with 10% fetal bovine serum (FBS, Gibco^®^, Grand Island, NY, USA) and 1% (*v/v*) penicillin/streptomycin (Sigma-Aldrich^®,^ Steinheim, Germany), maintained at a 37 °C and 95% humidified air with a 5% CO_2_ environment. Cells were cultured in monolayers in T25 cm^2^ flasks (ThermoScientific^®^, Waltham, MA, USA). Cell culture was observed daily, media was replaced every 3 days, and at 80% confluency cells were subcultured. In each passage, the culture media was removed, and the cells were carefully washed three times with phosphate-buffered saline solution (PBS, Sigma-Aldrich^®^, Steinheim, Germany) at room temperature. Then, the PBS was removed, and 0.25% trypsin-ethylenediaminetetraacetic acid (EDTA, Sigma-Aldrich^®^, Steinheim, Germany), was added and incubated at 37 °C for 4–5 min. Cell detachment was confirmed visually in an inverted phase contrast microscope (Zeiss^®^, Oberkochen, Germany). Culture media was added to stop enzymatic detachment, and detached cells were transferred into a 15 mL falcon tube. The cell suspension was centrifuged at 1600 rpm for 10 min to promote the formation of a pellet, which was later resuspended in 0.850 mL of fresh culture medium to reach a final volume of 1 mL. Then cells were cryopreserved or re-plated after determining their number and cell viability using the trypan blue exclusion cell assay and an automated counter (Fisher Scientific^®^, Waltham, MA, USA). All described procedures were performed under aseptic conditions in a flow chamber.

#### 2.1.1. Cryopreservation

For cell cryopreservation, an appropriate number of 2 mL cryovials was labeled (indicating the cell line, passage, date, cell numbers, and cryopreservation media). After automatic counting and determination of cell viability, the desired number of cells suspended in 1 mL of culture medium was transferred to each vial. After adding cryopreserving agent, dimethyl sulfoxide (DMSO—10% (*v/v*)), and more medium to fill the vials, they were capped and transferred into a freezing container with isopropyl alcohol (ThermoScientific^®^, Waltham, MA, USA) for slow freezing (−1 °C/minute) at −80 °C for 3 days. For longtime storage, vials were transferred to liquid nitrogen storage containers (−196 °C) (Wharton^®^, Philadelphia, PA, USA).

#### 2.1.2. Colony Formation Assay

The colony formation unit (CFU) assay evaluates the ability of the cell line to form colonies from a single cell. Cells at 80% confluency were passed and seeded into 6-well plates (Orange Scientific^®^, Braine-l'Alleud, Belgium) at densities of 300 and 500 cells/well, in triplicate, for all cell lines. The plates were incubated for 14 days under standard conditions with daily monitorization to confirm the development of colonies with the exception of the MKN45 cell line that was incubated for 21 days. After 14 or 21 days, respectively, the medium was removed, and the cells were stained with 0.5% (*v/v*) crystal violet (CV) for 15 min at room temperature, and the number of colonies was determined. Only visible colonies were counted (>0.5 mm in diameter and without overlapping). 

#### 2.1.3. Wound Healing Assay

To determine the invasive and migratory capacity of the cancer cells, cell mobility was assessed by an in vitro wound healing assay. MKN28 and MKN45 cells (5 × 10^4^ cells/well) and AGS cells (1 × 10^4^ cells/well) were seeded onto 24-well plates, in triplicate for each cell line, to grow to full confluence in a monolayer for 72 h. Then, on the day before the scratch, the medium was changed to RPMI without FBS. A sterile 20–200 μL pipette tip was held vertically to make a scratch along the central area in each well. The detached cells and debris were removed by washing with 500 μL of PBS twice. Afterward, 500 μL of fresh medium was added and the plates incubated for 48 h to enable cells to migrate and invade the cell-free scratch. The scratch closure was monitored, and the progression registered at 0 h, 24 h, and 48 h at a magnification of 300×. Wound area closure was automatically quantified using ImageJ© software v.1.53t (NIH, Bethesda, MD, USA), measuring the gap size and tracking the gap width by drawing lines along the leading edges of each cell front. The results are presented as percentage of wound closure. 

#### 2.1.4. Senescence-Associated β-Galactosidase Assay

To perform the β-galactosidase assay, 6000 cells/cm^2^ were seeded onto 96-well plates until reaching total confluence. At 24 h, 48 h, and 72 h, the culture medium was removed and, after washing with PBS, the cells were incubated with 200 μL of β-galactosidase reagent (ThermoFisher Scientific^TM^), for 30 min at 37 °C, and then the absorbance was read at 405 nm using a microplate photometer (ThermoScientific^TM^, Waltham, MA, USA). The results are presented as optical density (OD), also referred to as absorbance.

#### 2.1.5. Cytogenetic Analysis

The cell karyotype of all cell lines was evaluated to determine chromosomal stability in terms of chromosome number and the occurrence of neoplastic changes. Cells were expanded, and once they were in the exponential growth phase and approaching 80% confluency, the cells were treated with 10 μg/mL colcemid solution (Gibco^®^, Grand Island, NY, USA) for 2–4 h at 37 °C to arrest the cell cycle in metaphase. Cells were then harvested and suspended in a hypotonic solution (potassium chloride: KCl 0.075 M, 15 min at 37 °C). After centrifugation (1700 rpm, 7 min), cells were fixed (6:1 methanol to acetic acid), centrifuged, and resuspended in 3:1 methanol to acetic acid. Cell smears were prepared onto glass slides, air dried, and stained with Giemsa (4%, 6 min) according to a routine laboratory procedure. Karyotyping was performed according to the International System for Human Cytogenic Nomenclature [30].

### 2.2. Immunocytochemistry

The three cell lines were submitted to immunocytochemical (ICC) analysis to detect specific antigens. Cells were maintained in culture until they reached 70–80% confluence, and then enzymatic detachment was performed with 0.25% trypsin-EDTA solution, and a paraffin cytoblock (GmbH^®^, Germany) was prepared. Sections of 2 µm were cut, deparaffinized, dehydrated, and subjected to immunocytochemical analysis. The slides were placed for 10 min in xylene solution, and subsequently the slides were up and down 5 times in xylene solution, 100% alcohol, 96% alcohol, and 70% alcohol and washed in water for 5 min. Three antigen retrievals were used. In the retrieval solution/water bath, the slides were placed in 10% retrieval solution and incubated in a water bath at 100 °C for 30 min and cooled down for 10 min. In the microwave/Extran 0.5% Extran was boiled and the slides incubated in the microwave for 10 min. With the pepsin method, the slides were incubated 30 min/37 °C in a 4% pepsin solution, and then the solution was discarded; TBS was added and incubated 3 min in the freezer to stop the reaction. Slides were subjected to immunocytochemical analysis using a Novolink™ Max Polymer Detection Systems (Leica Biosystems^®^, Nußloch, Germany) kit according to the manufacturer’s instructions. Information on the primary antibodies and antigen retrieval methods used is presented in Table 1. Antibodies were selected to detect the presence of markers of epithelial (AE1/AE3, E-cadherin, EpCam), mesenchymal (Vim), vascular (CD31), mast/stem cell (C-kit), neuroendocrine (synaptophysin), and leucocyte (CD18) origin. Negative controls were performed by replacing the primary antibody with another antibody of the same immunoglobulin isotype. Sections were also processed for routine staining with hematoxylin and eosin (H&E). Samples were observed, scored, and photographed using an Eclipse E600 microscope (Nikon^®^, Tokyo, Japan). For the different markers, the immunoexpression levels was scored according to the intensity of labelling (0, negative; +, weak; ++, moderate; and +++, strong). If distinct cytoplasmic and/or membrane staining was detected in at least 5% of the total cell populations, immunoreactivity was considered positive.

### 2.3. In Vitro Drugs Protocol

The potential of the antineoplastic drug 5-FU and three repurposing drugs (NAT, NTZ, and BZT) in the GC cell lines was analyzed. Cells were treated with 5-FU in concentrations ranging from 0.1 to 100 μM for 48 h, and cell survival was evaluated by MTT. Based on these results, a dose–response curve was obtained, and the half maximal inhibitory concentration (IC_50_) value was calculated. IC_50_ values > 100 μM were not considered.

### 2.4. Cell Viability Assay

To evaluate the chemotherapy pharmacodynamics, the three cell lines (AGS, MKN45, and MKN28) were exposed to the following drugs: (1) 5-FU, (2) natamycin, (3) nitazoxanide, and (4) benztropine. Cell proliferation/viability was evaluated as mitochondrial metabolic activity performing an MTT reduction assay. To perform the MTT assay, cells in the logarithmic growth phase were seeded onto 96-well plates at a density of 5 × 10^3^ cells/well, with a final volume of 200 μL per well and incubated overnight, in a 37 °C incubator with 5% CO_2_ and humidified atmosphere, to promote cellular adhesion to the plate. The drugs were aseptically dissolved in the culture media (1:1000) in different concentrations (0.1, 1, 10, 25, 50, and 100 μM) and applied to the cells for 24 and 48 h. For each time point, the culture medium was removed, and, with the chamber light off, 100 μL of MTT was added to each well. Then, the cells were incubated for 2 h at 37 °C, protected from light. Finally, 100 μL/well of DMSO was added after MTT solution removal, and absorbance was measured at 570 nm using a microplate reader. 

Afterward, 5-FU and NTZ were tested in combination on MKN28 cells. The IC_50_ of 5-FU (12.41 μM) was combined with the IC_50_ of NTZ (6.713 μM) or increasing concentrations of the same drug (0.1, 1, 10, 25, 50, and 100 μM). After 48 h, the MTT assay was performed as explained above, and the cell viability results were obtained.

### 2.5. Statistical Analysis

The data presented are, at least, from three independent experiments. Data are expressed as mean ± standard deviation (M ± SD). The results were analyzed using one-way ANOVA, followed by a Student *t*-test when comparing the control and treated cells from one cell line. Differences were considered statistically significant when *p* ≤ 0.05 for a confidence level of 95%. All the analyses were performed using the software GraphPad Prism version 8 (GraphPad Software, CA, USA). Significant results were presented using the symbol (*). * Statistically significant vs. control at *p* < 0.1. ** Statistically significant vs. control at *p* < 0.01. *** Statistically significant vs. control at *p* < 0.001. **** Statistically significant vs. control at *p* < 0.0001.

## 3. Results

### 3.1. Cell Line Characterization

#### 3.1.1. Morphology Features

**Figure 2:**
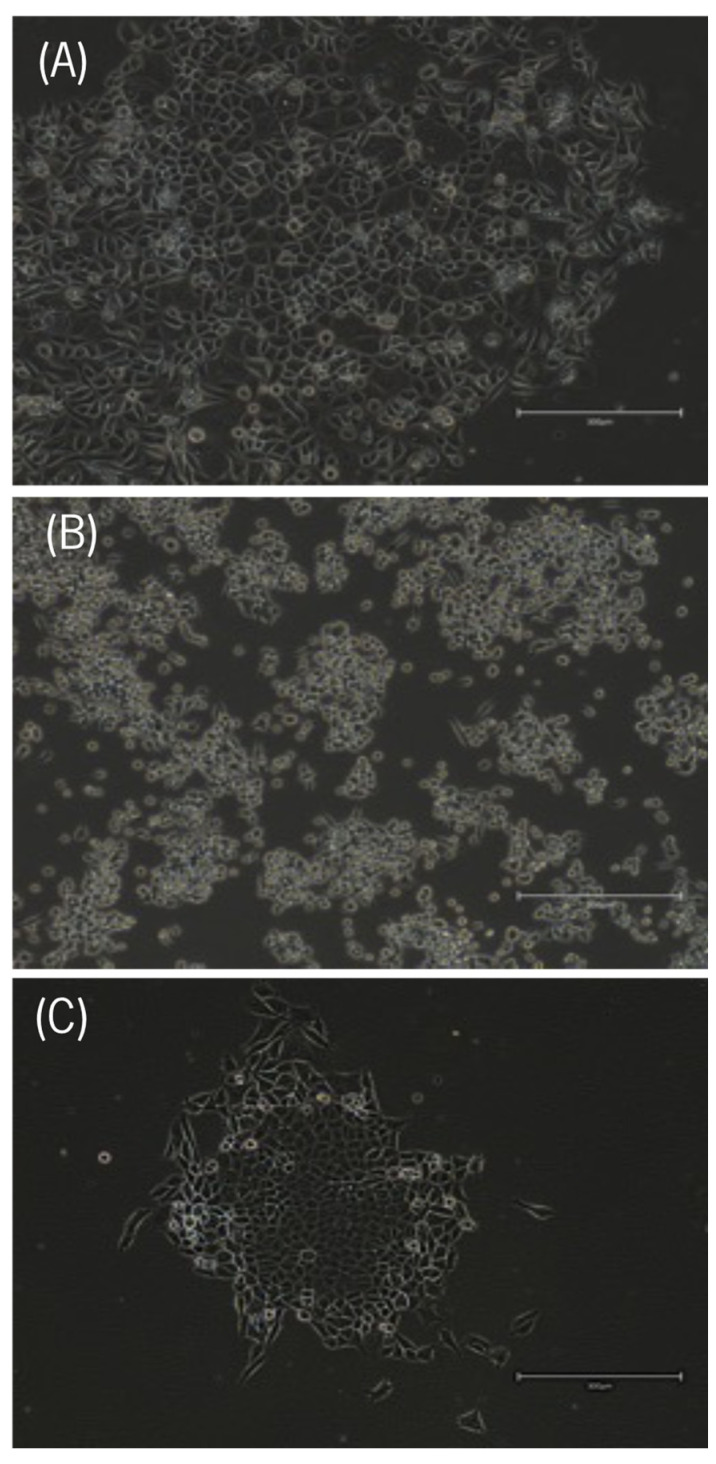
Morphology of the cell lines under study. (**A**) AGS cells with 90% confluence (passage 49); (**B**) MKN45 cells with 80% confluence (passage 16); and (**C**) MKN28 cells with 50% confluence (passage 4). Magnification, 20×; scale bars, 300 μm.

Almost all cell lines showed epithelial morphology when observed under light microscopy and after being cultured under the same conditions (Figure 2).

The analysis revealed that all cells showed clear plastic adhesion. Additionally, they mainly presented polygonal or "epithelial-like" morphologies, demonstrating intercellular adhesion and often forming cell groups with variable sizes (Figure 2). Most of the MKN45 cells appeared oval to spindle-shaped and grew in monolayers. Amongst all the cell lines, MKN45 presented the longest adhesion time and the lowest cell growth and proliferation rate.

#### 3.1.2. Colony Formation Assay

**Figure 3:**
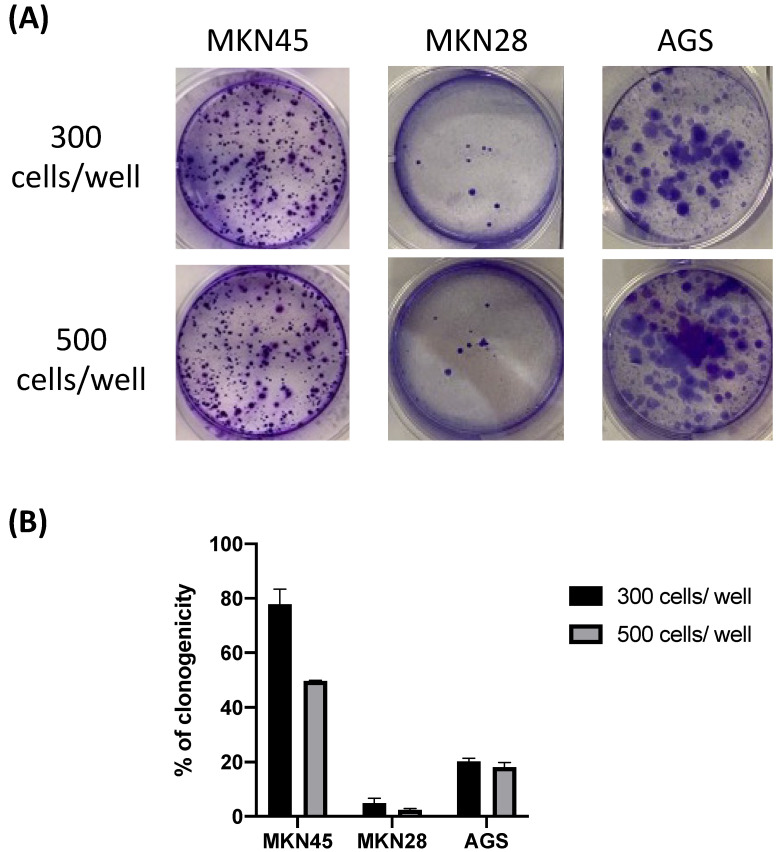
Results of the colony formation assay of MKN45, MKN28, and AGS cell lines performed 14 days after the seeding in cases of MKN28 and AGS and 21 days after seeding in the case of MKN45. Macroscopic visualization of the crystal violet-stained colonies (**A**) and percentage of colonies formed (**B**).

Two weeks after seeding (i.e., 14 days), crystal violet-stained colonies were visualized in all cell lines, with the exception of MKN45, which took 21 days to grow. The ability to form colonies was lower for AGS and MKN28 cells when compared with the MKN45 cells at both densities. Indeed, regardless of the density, MKN28 cells presented the lowest percentage of clonogenicity. The results of the colony formation assays are shown in Figure 3. 

#### 3.1.3. Wound Healing Assay

**Figure 4:**
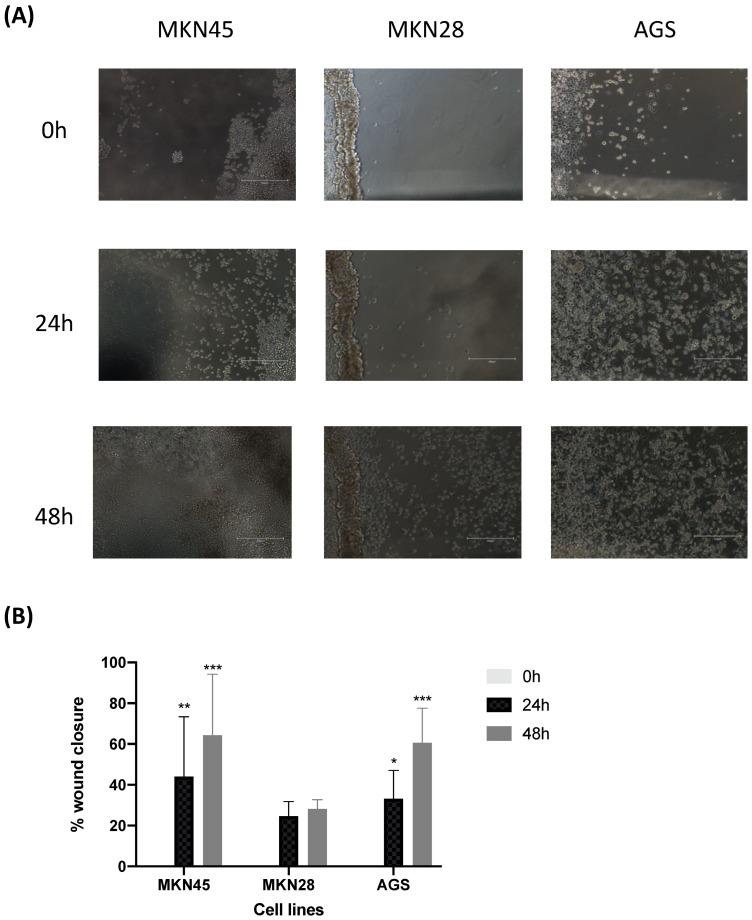
Results of MKN45, MKN28, and AGS cell lines by the wound healing assay. (**A**) Representative images of the different cell lines’ migration abilities with different time points (0 h, 24 h, and 48 h); (**B**) area quantification of the different cell lines in the different time points (0 h, 24 h, and 48 h). Values are expressed as percentages of control and represent means ± SEM. Each experiment was conducted three times independently (*n* = 3); * statistically significant vs. control at *p* < 0.05. ** Statistically significant vs. control at *p* < 0.01. *** Statistically significant vs. control at *p* < 0.001; scale bars, 300 μm.

Each cell line displayed different wound closure dynamics (Figure 4). In the cell scratch assay, the AGS cell line was the one that showed a faster and more effective migration ability. Nevertheless, with the exception of MKN28, an almost complete scratch closure was achieved in all lines after 48 h. 

The AGS cell line was shown to have significant differences at 24 h and 48 h from the start of scratching. At 24 h, the MKN28 cell line displays fast wound closure; however, it seemed to decrease and stagnate at 48 h, not making much difference from that observed at 24 h, with no significant differences for any time point in this cell line.

#### 3.1.4. Senescence-Associated β-Galactosidase Assay

**Figure 5:**
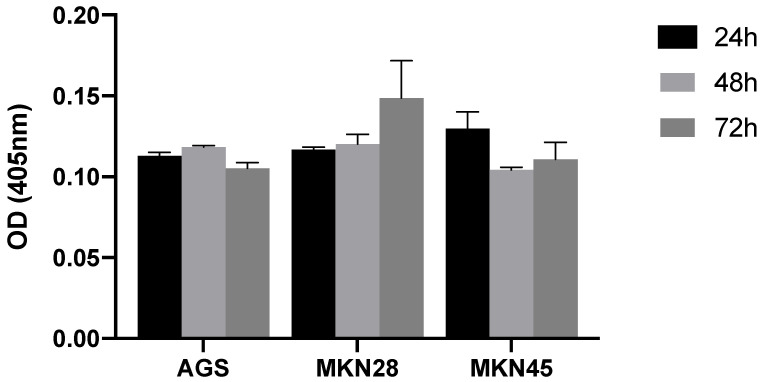
Results of MKN28, AGS, and MKN45 cell line senescence in β-galactosidase assay at different time points (24 h, 48 h, and 72 h). No significant association was found. OD, optical density.

A cellular senescence assay was also performed, and the respective results are shown in Figure 5. Our results showed that MKN28 was the most stable cell line in the first two time points (24 h and 48 h) and presented a higher senescence value at 72 h in comparison with the other cell lines. Regarding AGS, cell death was more evident at the second time point (48 h) and senescence decreased at 72 h. In fact, AGS presented the lowest senescence at this time point, which means that this line was more resistant to the excess of confluency. Analyzing the MKN45 cell line, the cells had more senescence at 24 h. In the other two time points, senescence decreased, showing more stability of this line within this time.

#### 3.1.5. Cytogenetic Analysis

In order to identify chromosomal abnormalities in GC, conventional karyotyping was performed in all cell lines. The frequency of normal and aneuploid cells detected in the different cell lines evaluation is depicted in Table 2.

The karyotype analysis showed that all the cell lines were aneuploid and were constituted by a very low percentage of cells with a normal karyotype (13.3% and 0% of cells with normal karyotypes for AGS, MKN28, and MKN45, respectively).

### 3.2. Immunocytochemistry

**Figure 6:**
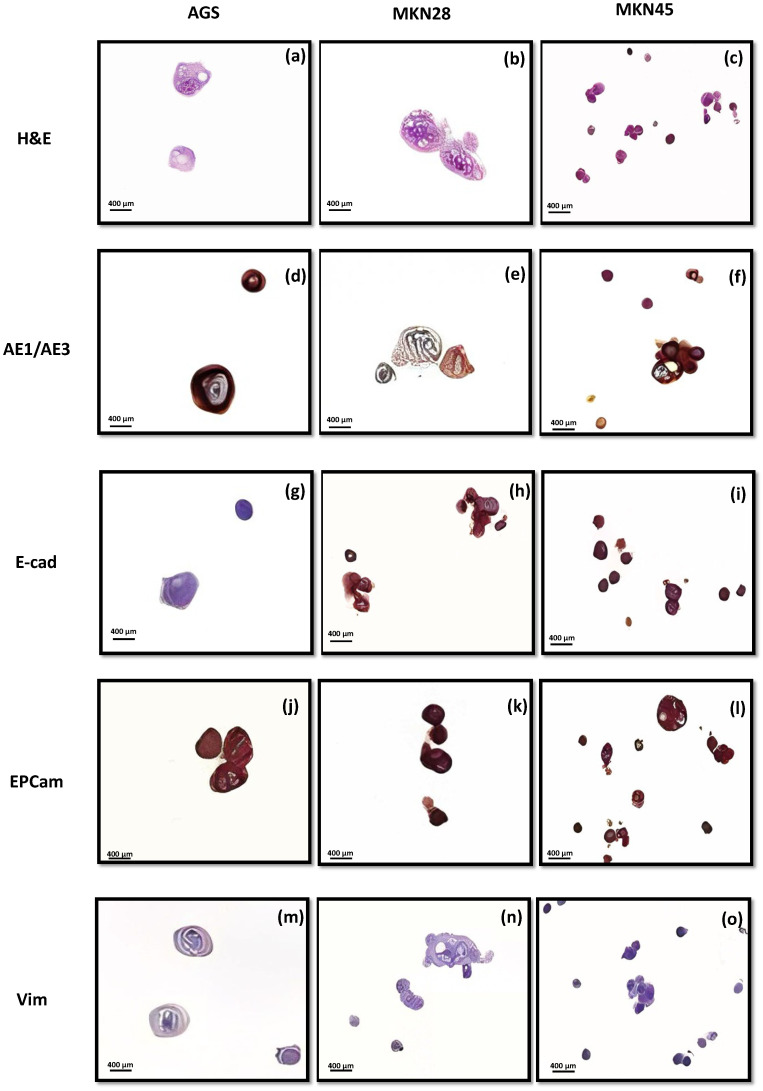
Immunocytochemistry of AGS, MKN28, and MKN45 cell lines: (**a**), (**b**), and (**c**) microscopic examination revealing different characteristics of every cell line (H&E); (**d** (+) **e** and **f** (+++)) for AE1/AE3; (**g** (0), **h** (+), **i**(+++)) for E-cadherin; (**j**), (**k**), and (**l**) positive immunoexpression of EPCam (++); (**m**, **n,** and **o** (0)) for vimentin. Magnification, 40×; scale bars, 400 μm.

Regarding H&E, all the cell lines were constituted of round to oval cells with marked pleomorphism (cells with different sizes). The AGS neoplastic cells commonly exhibited cytoplasm vacuoles. The MKN45 neoplastic cells presented a predominant plasmacytoid morphology. The immunocytochemistry revealed cytoplasm immunoexpression of the epithelial markers AE1/AE3 and EpCam in all cell lines evaluated. However, the privation of E-cadherin expression was observed in the AGS cell line (Figure 6g). All cell lines did not express vimentin. 

All the cell lines tested were also negative for the remaining antibodies (C-kit, CD31, synaptophysin and CD18) (Supplementary Figure S1).

### 3.3. In Vitro Drug Results

After characterizing the cell lines, the evaluation of chemotherapy pharmacodynamics and the in vitro response of the different chemotherapeutic protocols in GC cell lines (MKN28, MKN45, and AGS) were investigated. Cells treated with vehicle (culture medium with 0.1% of DMSO) were used as control. 

#### 3.3.1. MTT Results of the Different Cell Lines with 5-FU as Single Agents

**Figure 7:**
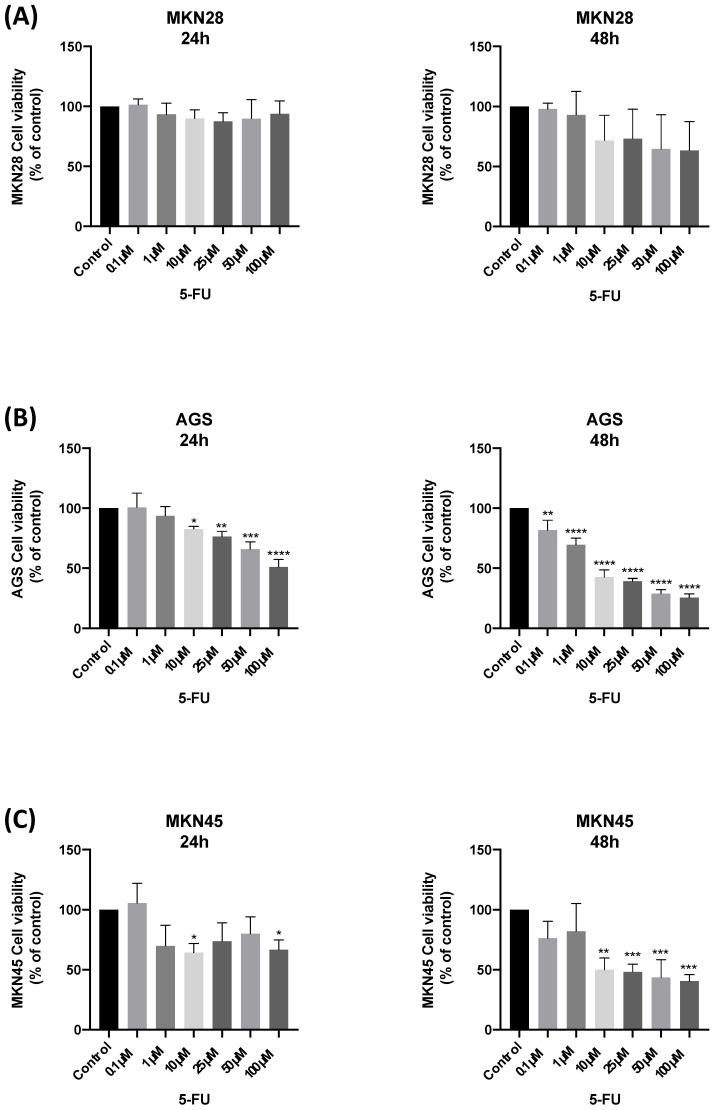
Viability of GC cells incubated with 5-FU. Cultured cells were seeded in 96-well plates and treated with 5-FU (0.1–100 μM) for 24 and 48 h. **(A**) The effect of 5-FU on MKN28 cell viability at 24 h and 48 h. (**B**) The effect of 5-FU on AGS cell viability at 24 h and 48 h. (**C**) The effect of 5-FU on MKN45 cell viability at 24 h and 48 h. Values are expressed as percentages of control and represent means ± SEM. Each experiment was done three times independently (*n* = 3); * statistically significant vs. control at *p <* 0.05. ** Statistically significant vs. control at *p* < 0.01. *** Statistically significant vs. control at *p <* 0.001. **** Statistically significant vs. control at *p <* 0.0001.

The results of the MTT assay for 5-FU in MKN28, AGS, and MKN45 cell lines are shown in Figure 7. 

The results of the MTT assay in the MKN28 cell line showed no significant differences at any time point (24 h and 48 h).

The results of the MTT assay in the AGS cell line showed that at 24 h, only concentrations above 10 μM exhibited significant differences compared to the control, requiring 20 μM of the antineoplastic drug to kill 50% of the cells. At the 48h time point, from a concentration of 0.1 μM, significant differences were already observed, with less than 2 μM of 5-FU being necessary to reduce 50% of AGS viability.

The results of the MTT assay in the MKN45 cell line showed that at 24 h, only two concentrations (10 μM and 100 μM) presented significant differences relative to the control. At 48 h, from a concentration of 10 μM, significant differences were already observed, with less than 2 μM of 5-FU being necessary to reduce 50% of MKN45 cells’ viability. 

The MTT assay for 5-FU treatment demonstrated a lack of efficiency after 24 h for MKN28 and MKN45 cell lines (Figure 7A,C), with some effect for AGS cells (Figure 7B). On the other hand, this assay demonstrated a solid cytotoxicity effect of this drug in AGS and MKN45 cell lines after 48 h (Figure 7B,C). The results for the AGS cell line revealed significant activity of 5-FU at concentrations above 10 μM after 24 h and 0.1 μM after 48 h (Figure 7B). The cells displayed a mild response to the cytotoxic effect of 5-FU, with less than 2 μM killing nearly 50% of cells (Table 3). Treatment of MKN45 cells was effective at concentrations above 10 μM, resulting in an IC50 value of less than 2 μM (Table 3). In MKN28, this drug was not shown to have a very cytotoxic effect on the cells, either at 24 h or 48 h (Figure 7A). These results confirm the anticancer activity of 5-FU in AGS and MKN45 cancer cell lines and justify its use in GC treatment.

#### 3.3.2. MTT Results with Repurposed Drugs as Single Agents

The potential antitumor effects of different repurposed drugs as single agents, namely, NAT, NTZ, and BZT in MKN28, AGS, and MKN45 GC cells were evaluated. The three cell lines were treated with increasing concentrations of each drug, from 1 to 100 μM, to investigate the viability of the cells 24 and 48 h after treatment.

**Figure 8:**
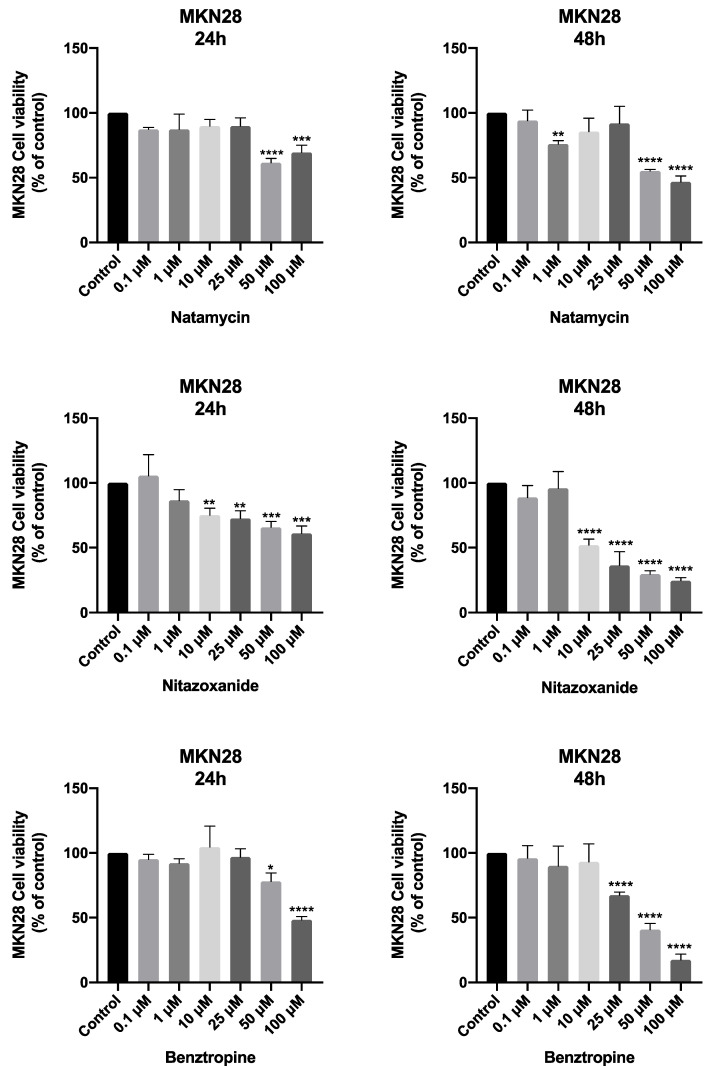
Viability of MKN28 GC cells incubated with different repurposed drugs alone. Cultured cells were seeded in 96-well plates and treated with each drug alone (0.1–100 μM) for 24 and 48 h. Values are expressed as percentages of control and represent means ± SEM. Each experiment was conducted three times independently (*n* = 3); * statistically significant vs. control at *p <* 0.05; ** statistically significant vs. control at *p <* 0.01; *** statistically significant vs. control at *p <* 0.001; **** statistically significant vs. control at *p <* 0.0001.

**Figure 9:**
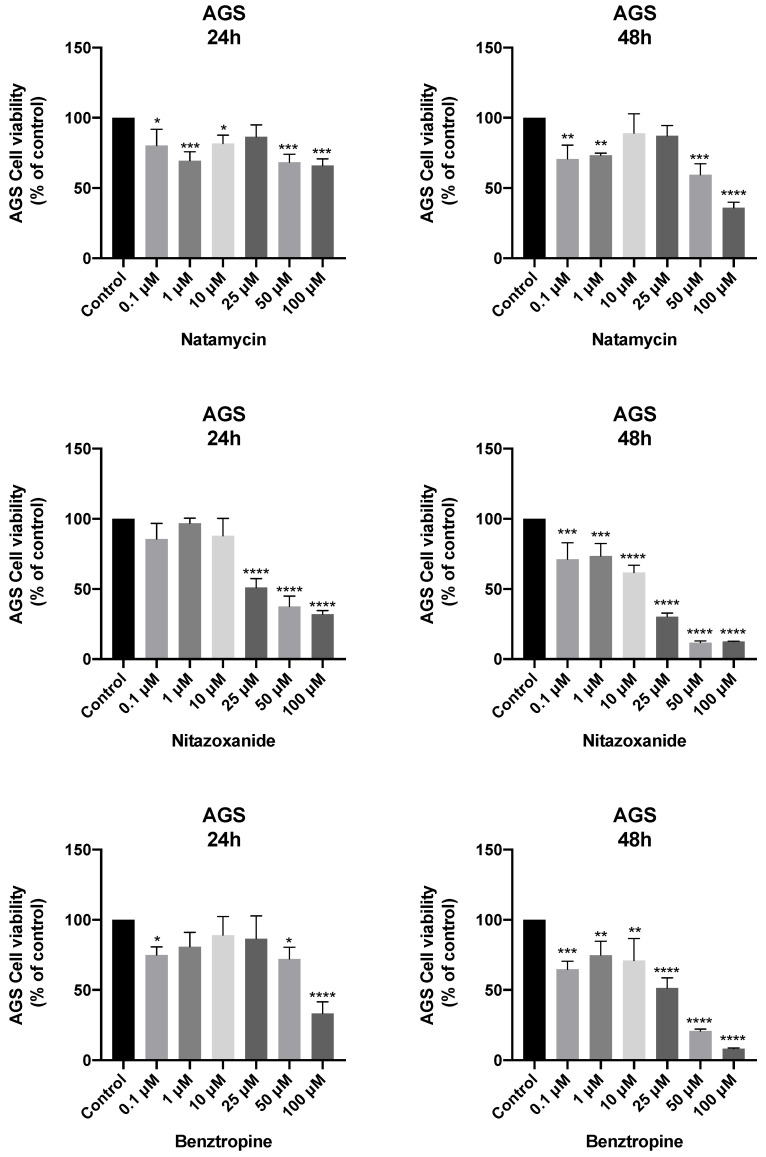
Viability of AGS GC cells incubated with different repurposed drugs alone. Cultured cells were seeded in 96-well plates and treated with each drug alone (0.1–100 μM) for 24 and 48 h. Values are expressed as percentages of control and represent means ± SEM. Each experiment was conducted three times independently (*n* = 3); * statistically significant vs. control at *p <* 0.05; ** statistically significant vs. control at *p <* 0.01; *** statistically significant vs. control at *p <* 0.001; **** statistically significant vs. control at *p <* 0.0001.

Based on these results, NTZ displays a significant effect in MKN28 cells (Figure 8). The dose–response curve for NTZ at 24 h and 48 h revealed an IC_50_ value of 4.42 μM and 6.71 μM, respectively (Table 3). The rest of the repurposed drugs (NAT and BZT) had a cytotoxic effect on the MKN28 cells only at the highest concentrations (50 μM and 100 μM) (Figure 8). The results of the MTT assay for the AGS cell line are shown in Figure 9.

Based on these results, NTZ and BZT displayed significant effects in AGS cells. Treatment with BZT at doses above 0.1 μM for 48 h had a strong effect on cell viability (Figure 9), resulting in an IC50 value of 5.8 μM (Table 3). All the concentrations of NTZ above 25 μM for 24 h and 0.1 μM for 48 h showed a strong effect on cell viability with an IC50 less than 3 μM for 48 h (Table 3). In AGS cells, the drug NAT demonstrated a lack of efficiency in reducing cell viability (Figure 9). 

**Figure 10:**
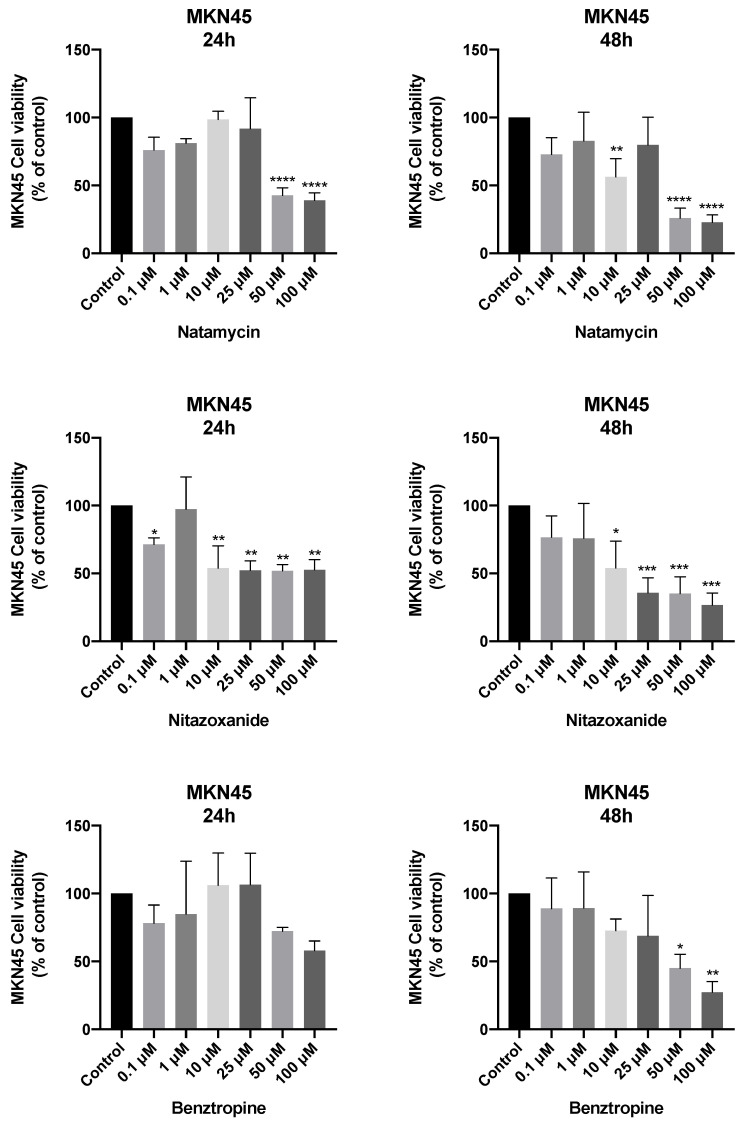
Viability of MKN45 GC cells incubated with different repurposed drugs alone. Cultured cells were seeded in 96-well plates and treated with each drug alone (0.1–100 μM) for 24 and 48 h. Values are expressed as percentages of control and represent means ± SEM. Each experiment was conducted three times independently (*n* = 3); * statistically significant vs. control at *p <* 0.05; ** statistically significant vs. control at *p <* 0.01; *** statistically significant vs. control at *p <* 0.001; **** statistically significant vs. control at *p <* 0.0001.

The MTT assay results for the MKN45 cell line are shown in Figure 10. The MTT assay for MKN45 cells demonstrated a strong cytotoxic effect of NAT and NTZ. NAT showed significant anti-tumor effects at concentrations above 50 μM for 24 h and 48 h (Figure 10), with an IC50 value of 33.9 μM and 6 μM for 24 h and 48 h, respectively (Table 3). The cytotoxic effects of NTZ (Figure 10) were significant even at 24 h of treatment, with 3.7 μM and 1.9 μM causing death of more than 50% of cells for 24 h and 48 h, respectively (Table 3).

For 24 h, only NTZ was shown to have advantageous values of IC50; the remaining drugs had very high values. At 48 h, NTZ was again the drug with the lowest IC50 values and thus the only active drug in all cell lines. Additionally, NAT was shown to have an effect on the MKN45 cell line, and BZT on the MKN45 and AGS cell lines.

#### 3.3.3. MTT Results with Combined Drugs

**Figure 11:**
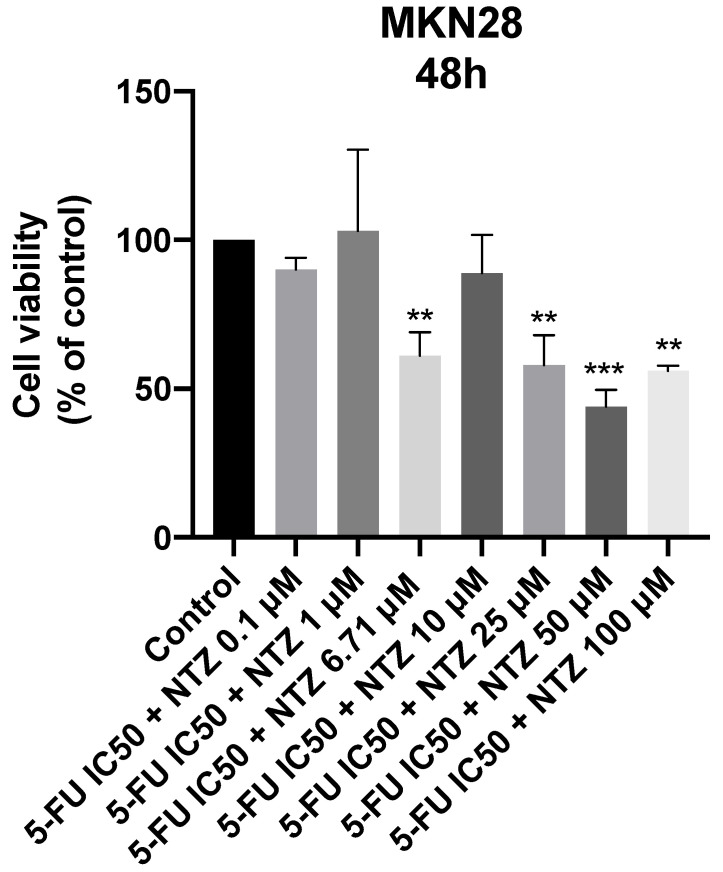
Cell viability of MKN28 cells treated with the combination of 5-FU and nitazoxanide. Values are expressed as percentages of control and represents means ± SEM. Each experiment was done three times independently (*n* = 3); ** statistically significant vs. control at *p <* 0.01; *** statistically significant vs. control at *p <* 0.001.

After finding the best candidates for drug repurposing in GC therapy and their IC_50_ values, the co-treatment of 5-FU with NTZ in the MKN28 cell line was evaluated, since it was the GC cell line with half the IC50 value of NTZ compared to 5-FU. 5-FU was selected as it was the reference anticancer drug. NTZ was chosen for being the only repurposed drug active in all cell lines in low concentrations. The results of the MTT assay for the combination of 5-FU and NTZ are shown in Figure 11.

Co-treatment of MKN28 cells with 5-FU and NTZ was shown to have more cytotoxicity than 5-FU alone (Figure 7) but did not increase cytotoxicity compared to NTZ alone (Figure 8).

## 4. Discussion

GC remains one of the most common cancers worldwide, and most cases of GC are asymptomatic, with many only being diagnosed at advanced stages. 

Despite massive efforts over the years to improve cancer therapies, the high mortality rate is mainly due to the development of resistance to chemotherapy, which is one of the biggest challenges in any type of cancer treatment. As a result, there has been a focus on finding novel therapeutic strategies. The development of new drugs has some weaknesses, as it is time-consuming, costly, and requires clinical trials that frequently fail in the early stages. One way to overcome these drawbacks is drug repurposing [31,32].

Since the main objective of this investigation was to evaluate the in vitro response of different chemotherapeutic protocols in GC cell lines, morphological, biochemical, and genetic characterizations of MKN28, AGS, and MKN45 cell lines were carried out. Although all cell lines were derived from gastric carcinoma, they harbor different genetic backgrounds.

Between 1 and 2 weeks after cell thawing, epithelial-like morphologies were observed in MKN28 and AGS cells. These results are in accordance with previous studies [33,34,35]. MKN45 cells have been shown to have a spindle and oval-shaped form, and the adhesion time was longer due to their monolayer growth and clumps in suspension [36]. According to Walen, the messy growth and development of agglomerates in a cell line are triggered by the loss of contact inhibition or density-based growth control [37].

The colony formation unit assay is an in vitro cell survival test that measures the capacity of a single cell to divide and give rise to a colony through successive divisions. Thus, this method reflects the proliferative capacity of cells and their ability to generate new epithelial colonies from single cells seeded at low densities [38,39]. Clonogenic assays are widely used in cancer investigation because clone formation is interpreted as a characteristic of cancer cells with tumor-initiating capabilities. This assay has become a standard tool in cancer research to evaluate cell growth and cytotoxic or genotoxic effects of various agents with potential clinical applications [40]. In the present study, all cell lines were able to form colonies, MKN45 being the cell line capable of originating more colonies from single cells seeded at 300 and 500 cell/well density. On the other hand, this cell line is also the slowest to grow and start to grow in colonies. Furthermore, it should also be noted that clonogenicity is higher for lower sowing densities, probably due to competitive inhibition phenomena. The clonogenicity results are consistent with the results found in the literature [41,42,43,44]. This method shows that cancer stem cells can survive and form colonies in an anchorage-independent culture model.

A wound is defined as damage to or disruption of the anatomic structure and function of the underlying normal tissue [45]. In addition, cell migration is linked to many physiological and pathological processes related to embryogenesis, wound repair, and cancer metastasis. An important aspect of metastasis is the ability of cancer cells to invade surrounding tissues, which is mainly determined by cell mobility [46]. Therefore, the wound healing assay is widely applicable since it is a simple, non-expensive, and extremely reproducible method to study cell migration of cancer in vitro [4]. The results demonstrate that the ability to migrate varies according to the cell line tested. MKN45 cells have the fastest wound closure, similar to AGS cells. These results about the similar wound closure between AGS and MKN45 cell lines agree with other studies [47,48]. Importantly, the gaining of migration properties is essential during tumor progression and metastasis, which suggests that all of these cell lines may have metastasis features [49].

Senescence corresponds to cellular aging with resultant changes in the cell division cycle. Cellular senescence has been described as a state of permanent arrest of cell division after a stage of successive proliferation of normal diploid cells in vitro [50]. This can be determined by quantifying the activity of β-galactosidase, which makes it possible to label senescent cells in a simple, rapid, and effective way [51]. Regarding this assay, MKN28 was the most stable cell line at 24 h and 48 h, with increased senescence at 72 h, evidencing a common behavior that occurs with the passage of time. Regarding the AGS cell line, the cell death was higher at 48 h and became stable after 72 h, which means this line can be kept in culture for a long time because it resists the excess of confluency. However, the results suggested that MKN28 is not a cell line that can be kept for a long time in culture because the cells started to die at 72 h. Analyzing the MKN45 cell line, the cells have more senescence at 24 h due to their slow adhesion, monolayer growth, presence, and single round cells or clumps in suspension.

The results concerning the karyotype analysis were as expected, since all cell lines present well-known malignant properties, which supports the thesis that chromosomal instability can be a persistent characteristic of cancer cells [52,53]. Motoyama et al. had already described that the MKN45 cell line was hypodiploid, a result that is in line with those obtained in this study [54].

Antibody cocktails for keratin such as AE1/AE3 were positive in the three GC cell lines, being in accordance with Krasinskas and Goldsmith who claimed that gastric adenocarcinomas are strongly positive for AE1/AE3, which is in line with their epithelial histogenesis [55].

In culture, AGS cells have lost expression of the epithelial-specific gene E-cadherin. The epithelial-to-mesenchymal transition (EMT) is an acute event in cancer metastasis, characterized by reciprocal loss of expression of epithelial cell-associated proteins, such as E-cadherin, and increased levels of mesenchymal markers, such as vimentin [56,57].

EPCam, an epithelial cell membrane molecule, was expressed in all cell lines. The increased levels of EPCam in GC and metastatic lesions indicate that this marker is a promising therapeutic target [58]. All the cell lines evaluated did not express any of the remaining immunomarkers. Since CD31 is considered a specific vascular marker [59], CD18 is expressed on tissue macrophages, dendritic cells, and eosinophils [60], and synaptophysin is a protein marker for neuroendocrine cells [61], these results were expected.

In this investigation, 5-FU was chosen as the reference anti-neoplastic drug, highlighting its wide use in GC treatment [62]. Based on the literature available and in the interests of this research, three drugs were selected and tested in MKN28, AGS, and MKN45 GC cell lines. A recent study stated that NAT suppresses the enzyme of the base excision repair (BER) mechanism in prostate cancer, decreasing tumor cell proliferation. This enzyme is over-expressed in several cancer types, including GC [20,21]. There are no studies about NAT in GC; however, it could be a potential drug to be investigated in the future. For instance, NTZ in colon cancer cells inhibits cell development, nuclear condensation, and DNA fragmentation and induces apoptosis [24]. In breast cancer cells, NTZ leads to tumor growth suppression and induces apoptosis [63]. NTZ is an anthelmintic with the ability to eradicate *H. pylori*, one of the risk factors for the development of GC. In addition, like other anthelmintics, NTZ has shown good anti-tumor activity in other types of digestive tract cancers [64]. Furthermore, this drug acts on the Wnt/β-catenin pathway, the expression of which is often aberrant in a large proportion of GC [25]. Despite BZT having never been tested in GC, Sogawa et al. stated that this drug acts on the dopamine transporter SLC6A3/DAT, which is overexpressed in several cancer types, including GC [29].

After evaluation with MTT, IC_50_ was determined for each drug and hour, and based on the best results, 5-FU and NTZ were selected for the drug combination. The results demonstrated that repurposing the drugs as single agents had the ability to decrease cell viability in all cell lines in a concentration-dependent manner. The cell viability of MKN28, AGS, and MKN45 cells treated with 5-FU resulted in concentration-dependent growth inhibition after 48 h. The drug NTZ, in the repurposing category, was the only active drug in all cell lines. The other repurposing drugs (NAT and BZT) demonstrated a lack of efficiency in reducing cell line viability after 24 h. After 48 h of treatment, these drugs showed some cytotoxic effects, namely, NAT on the MKN45 cell line and BZT on the AGS cell line.

As the cell lines have different histological origins, different results were expected for each cell line type. Based on the results, the drug NTZ seems to have a cytotoxic effect on both intestinal and diffuse GC. NAT was only shown to be cytotoxically active in the MKN45 cell line, which is derived from diffuse type GC. BZT was shown to have interesting results in that it was active in the AGS cell line, which is histologically derived from both GC types (diffuse and intestinal) but was not shown to have cytotoxicity in the remaining lines (MKN28 and MKN45), which are each derived from different GC types, namely, the intestinal and diffuse types.

After determining the anticancer potential of each repurposing drug in MKN28, AGS, and MKN45 GC cells, the drug with the best IC_50_ (NTZ) was selected and combined with the reference drug 5-FU for 48 h. This task aimed to evaluate if the combination of NTZ with the antineoplastic drug (5-FU) would enhance their cytotoxic effect on MKN28 cells. The results obtained proved that there is no synergetic effect of 5-FU and NTZ. In fact, NTZ displayed more cytotoxic effects than 5-FU alone. From literature research, studies that used an NTZ-based treatment for *H. pylori* identified and demonstrated a cure rate success higher than 80% [65,66,67]. This antibacterial effect of NTZ could then leave cells more vulnerable to the 5-FU effect, which could be the reason behind the stronger effect of their use when compared with 5-FU alone.

With the increasing resistance of various types of cancer, such as GC, to the reference drugs used for their treatment, it is important to find alternatives. The use of repurposing drugs as a treatment for GC has proven to be a promising area of research, having the potential to improve outcomes for patients with this type of cancer.

## 5. Conclusions

In the present investigation it was demonstrated that the use of repurposing drugs as single agents has the ability to decrease cell viability in three GC cell lines in a concentration-dependent manner. NTZ was the only repurposed drug that significantly reduced cell viability in all cell lines. The remaining repurposing drugs, NAT and BZT, demonstrated a lack of efficiency in cell viability reduction at 24 h. The cytotoxic effects of these drugs were only significant in concentrations above 50 µM after 48 h of treatment. Then, MKN28 cell line was treated with a drug combination of NTZ with the antineoplastic drug 5-FU. 

In general, the use of NTZ alone was the most promising drug for GC therapy, since this drug displayed more cytotoxic effects than either drug by itself. However, thorough studies are strongly recommended to evaluate in more detail the anticancer properties of this drug combination and further evaluate its action in animal models and eventually in clinical trials. Taken together, these results demonstrate that there is a great potential in the use of repurposed drugs and in their inclusion in combination schemes in order to promote and develop novel therapeutic strategies for GC.

Moreover, deeper mechanistic studies are strongly recommended to evaluate the anticancer properties of this drug combination and further confirmation on animal models and clinical trials. Taken together, these results demonstrate that there is great potential in the use of repurposed drugs for the treatment of GC. The inclusion of these drugs in combination schemes has also shown potential in order to promote and develop new therapeutic strategies for GC.

## Figures and Tables

**Table 1:** Antibodies used and conditions applied in the immunocytochemical analysis.

Antibody	Antigen Retrieval	Dilution	Manufacturer
E-cadherin	Microwave/Extran	1:50	Life Technologies
Vimentin	Retrieval solution/Water bath	1:500	Dako
EpCam		1:450	Invitrogen
C-kit			Dako
Cytokeratin AE1/AE3		1:1200	Invitrogen
CD31	Pepsin/Incubator	1:50	Dako
Synaptophysin	Retrieval solution/Water bath	1:150	Thermo Scientific
CD18		1:100	Antiserum

**Table 2:** Percentage of normal and aneuploid present in AGS (passage 49), MKN28 (passage 16), and MKN45 (passage 4) cell lines.

Cell Line	% Cells with Normal Karyotype (2n)	% Hypodiploid Cells	% Hyperdiploid Cells
AGS	13.3	43.3	13.3
MKN28	0	2	4
MKN45	0	56	0

**Table 3:** IC50 of reference and repurposed drugs in AGS, MKN28, and MKN45 GC cells.

24 h/IC50 μM				48 h/IC50 μM		
	AGS	MKN28	MKN45	AGS	MKN28	MKN45
5-FU	20.05	>100	>100	1.25	12.41	1.11
Natamycin	>100	25.66	33.58	39.57	35.36	6.02
Nitazoxanide	17.75	4.42	3.72	2.79	6.71	1.95
Benztropine	49.98	52.67	>100	5.76	30.71	20.95

## Data Availability

Not applicable.

## Supplementary Materials

The following supporting information can be downloaded at: https://www.mdpi.com/article/10.3390/biomedicines11030799/s1, Figure S1: Immunocytochemistry of neoplastic cells.
