# Supplementary material for: Repurposing Benztropine, Natamycin, and Nitazoxanide Using Drug Combination and Characterization of Gastric Cancer Cell Lines
Source: Biomedicines. 2023 Mar 6;11(3):799. doi: 10.3390/biomedicines11030799 (PMC10044866; doi:10.3390/biomedicines11030799)
Supplement: Supplementary file 1 [file biomedicines-11-00799-s001.zip › Supplementary Materials.pdf]

Supplementary Materials: Repurposing Benztropine, Natamycin and Nitazoxanide Using Drug Combination and Characterization of Gastric Cancer Cell lines

Eduarda Ribeiro [1,2,†], Diana Araújo [1,2,3,4,†], Mariana Pereira [1,2,5], Bruna Lopes [6,7,8], Patrícia Sousa [6,7,8], Ana Catarina Sousa [6,7,8], André Coelho [6,7,8], Alexandra Rêma [6,7,8], Rui Alvites [6,7,8], Fátima Faria [2], Cláudia Oliveira [9], Beatriz Porto [9], Ana Colette Maurício [6,7,8], Irina Amorim [2,3,4] and Nuno Vale [1,5,10,*]

1 OncoPharma Research Group, Center for Health Technology and Services Research (CINTESIS), Rua Doutor Plácido da Costa, 4200-450 Porto, Portugal
2 Departamento de Patologia e Imunologia Molecular Instituto de Ciências Biomédicas de Abel Salazar (ICBAS), Universidade do Porto, Rua de Jorge Viterbo Ferreira 228, 4050-313 Porto, Portugal
3 Institute for Research and Innovation in Health (i3S), Universidade do Porto, Rua Alfredo Allen, 208, 4200-135 Porto, Portugal
4 Institute of Molecular Pathology and Immunology, University of Porto (IPATIMUP), Rua Júlio Amaral de Carvalho, 45, 4200-135 Porto, Portugal
5 CINTESIS@RISE, Faculty of Medicine, University of Porto, Alameda Professor Hernâni Monteiro, 4200-319 Porto, Portugal
6 Departamento de Clínicas Veterinárias, Instituto de Ciências Biomédicas de Abel Salazar (ICBAS), Universidade do Porto (UP), Rua de Jorge Viterbo Ferreira, nº 228, 4050-313 Porto, Portugal
7 Centro de Estudos de Ciência Animal (CECA), Instituto de Ciências, Tecnologias e Agroambiente da Universidade do Porto (ICETA), Rua D. Manuel II, Apartado 55142, 4051-401 Porto, Portugal
8 Associate Laboratory for Animal and Veterinary Sciences (AL4AnimalS), Lisbon, Portugal
9 Laboratório de Citogenética, Instituto de Ciências Biomédicas de Abel Salazar (ICBAS), Universidade do Porto, Rua de Jorge Viterbo Ferreira, nº 228, 4050-313 Porto, Portugal
10 Department of Community Medicine, Health Information and Decision (MEDCIDS), Faculty of Medicine, University of Porto, Rua Doutor Plácido da Costa, 4200-450 Porto, Portugal
* Correspondence: nunovale@med.up.ptn; Tel.: +351-220426537
† These authors contributed equally to this work.

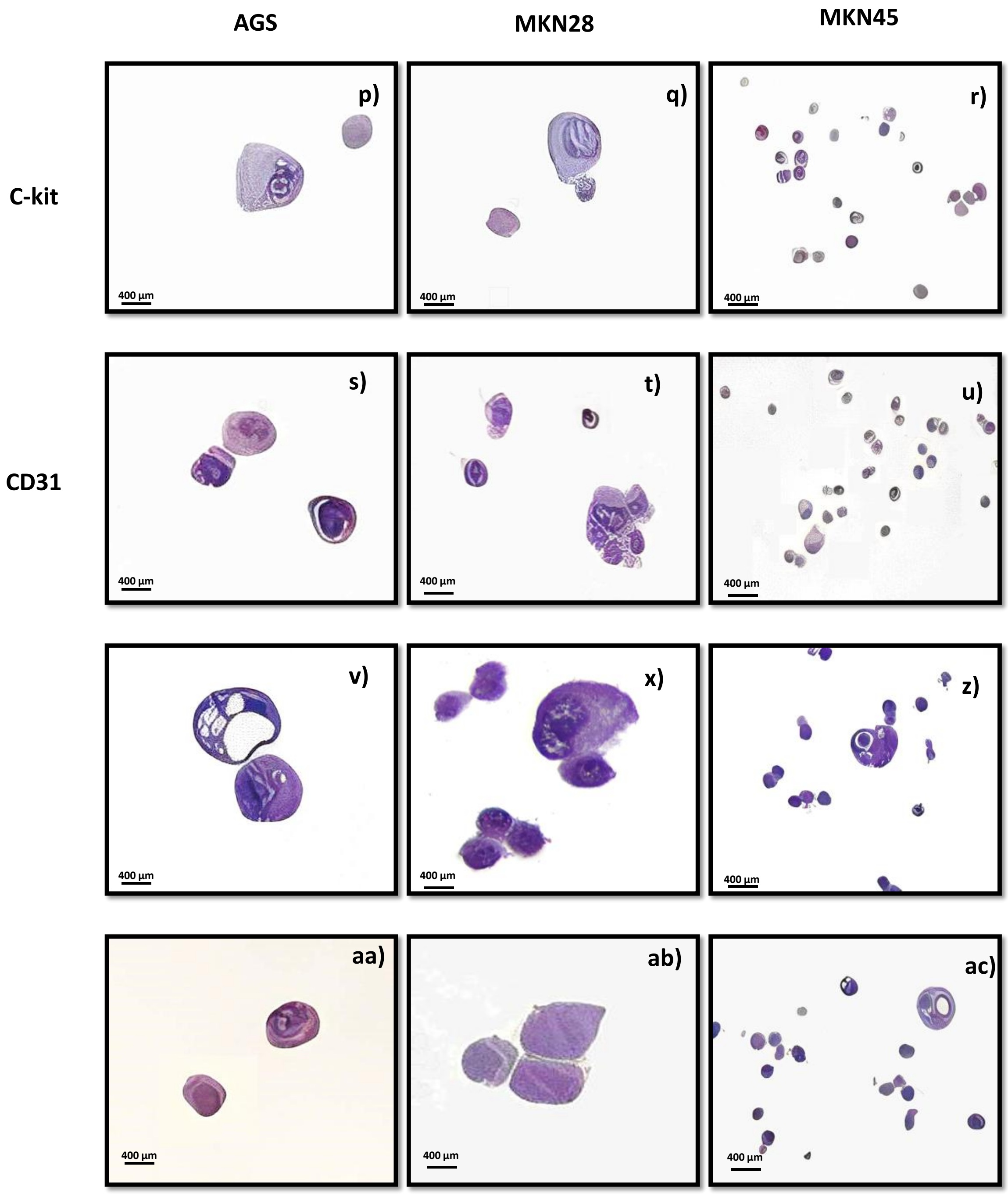

Figure S1. Immunocytochemistry of neoplastic cells: p), q) and r) negative immunoexpression of C-kit (0); s), t) and u) negative immunoexpression of CD31 (0); v), x), and z) negative immunoexpression of Synaptophysin (0); and aa), ab) and ac) negative immunoexpression of CD18 (0); Magnification, 40x; scale bars, 400 µm.
